# Design and Testing of Bistable Lattices with Tensegrity Architecture and Nanoscale Features Fabricated by Multiphoton Lithography

**DOI:** 10.3390/nano10040652

**Published:** 2020-03-31

**Authors:** Zacharias Vangelatos, Andrea Micheletti, Costas P. Grigoropoulos, Fernando Fraternali

**Affiliations:** 1 Department of Mechanical Engineering, University of California, Berkeley, CA 94709, USA; zacharias_angelatos@berkeley.edu; 2 Department of Civil and Computer Science Engineering, University of Rome Tor Vergata, 00133 Rome RM, Italy; micheletti@ing.uniroma2.it; 3 Department of Civil Engineering, University of Salerno, 84084 Fisciano SA, Italy; f.fraternali@unisa.it

**Keywords:** multiphoton lithography, direct laser writing, lattice metamaterials, tensegrity architecture, bistability, multistability

## Abstract

A bistable response is an innate feature of tensegrity metamaterials, which is a conundrum to attain in other metamaterials, since it ushers unconventional static and dynamical mechanical behaviors. This paper investigates the design, modeling, fabrication and testing of bistable lattices with tensegrity architecture and nanoscale features. First, a method to design bistable lattices tessellating tensegrity units is formulated. The additive manufacturing of these structures is performed through multiphoton lithography, which enables the fabrication of microscale structures with nanoscale features and extremely high resolution. Different modular lattices, comprised of struts with 250 nm minimum radius, are tested under loading-unloading uniaxial compression nanoindentation tests. The compression tests confirmed the activation of the designed bistable twisting mechanism in the examined lattices, combined with a moderate viscoelastic response. The force-displacement plots of the 3D assemblies of bistable tensegrity prisms reveal a softening behavior during the loading from the primary stable configuration and a subsequent snapping event that drives the structure into a secondary stable configuration. The twisting mechanism that characterizes such a transition is preserved after unloading and during repeated loading-unloading cycles. The results of the present study elucidate that fabrication of multistable tensegrity lattices is highly feasible via multiphoton lithography and promulgates the fabrication of multi-cell tensegrity metamaterials with unprecedented static and dynamic responses.

## 1. Introduction

The inexorable advances in materials science have been accomplished and conflated with the progress of additive manufacturing (AM) technologies over the recent years. Metamaterials comprised of lattice members are spatial periodic structures with unprecedented physical properties, mainly derived from the architecture of the repeated substructure, rather than the nature of the constituent materials. It is worth noting that extraordinary strength-to-weight and stiffness-to-weight ratios, frequency bandgaps, negative overall elastic moduli, negative mass density, auxeticity and solitary wave propagation represent characteristic and unconventional properties of such systems [1,2,3,4,5,6,7,8,9,10,11,12,13,14,15,16,17,18], as well as a multistable mechanical response [19,20,21,22,23,24,25,26,27,28]. However, the additive manufacturing of multi-cell realizations of mechanical metamaterials remains a challenge at present, due to the difficulty of reproducing the desired behaviors at small scales [8,9,29,30,31,32].

An intriguing category of lattice metamaterials is that of tensegrity frameworks: prestressed pin-connected lattices composed of bars (i.e., members loaded in compression) and cables (members loaded in tension), which typically feature internal mechanisms. Tensegrity structures exhibit different types of nonlinear static and dynamic responses, depending on their connectivity, geometry and state of selfstress [33,34,35]. Due to the complex nonlinear response of such structures, they have been employed to obtain different mechanical systems endowed with peculiar static and dynamic behaviors [15,16,17,18,36,37,38]. Some tensegrity modules can transcend from one stable state to two other stable states, either by changing the lengths of their elements or by changing the selfstress level [39]. This bistable response has been observed in some case studies at the macroscopic scale [40,41]. It can be expediently utilized to develop tunable, switchable and/or reconfigurable metamaterials, which are also able to support solitary wave dynamics [42].

**Figure 1:**
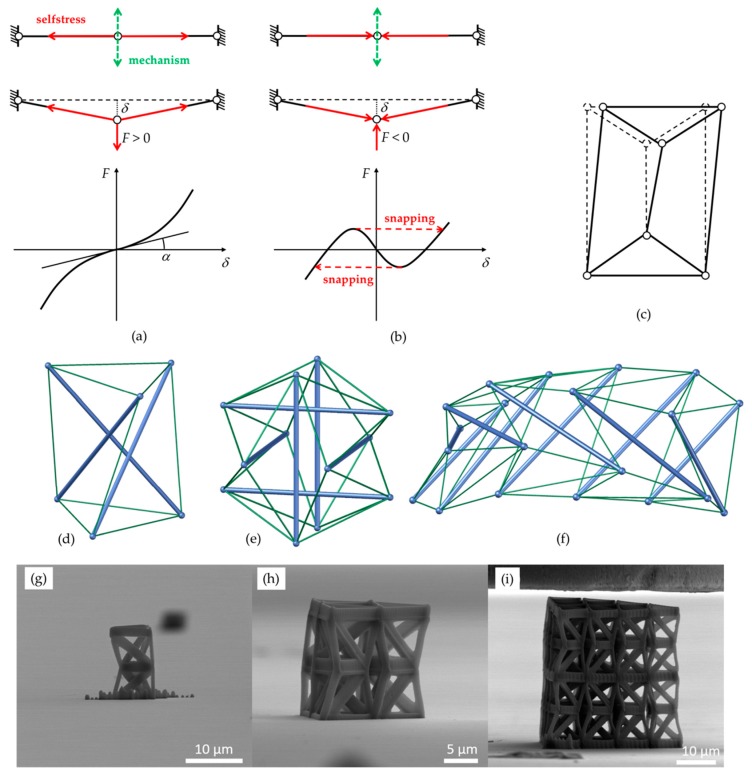
Illustration of the analyzed behaviors and fabricated structures. (**a**) Selfstress and mechanism in the prestress-stable two-element system (top). Deformed configuration under a vertical load and corresponding response (center and bottom). (**b**) Corresponding bistable system and bistable snapping response under the same vertical load. (**c**–**f**) Examples of prestress-stable systems with one seflstress state and one mechanism: (**c**) A two-dimensional system displaced along its mechanism; (**d**) triangular tensegrity prism; (**e**) expanded octahedron or tensegrity icosahedron; (**f**) an irregular *x*-tower. Fabricated geometries of structures: (**g**) individual unit cells, (**h**) arrays of three unit cells in one layer, (**i**) arrays of two layers with ten unit cells at each layer.

A remarkable property of tensegrity structures, which was first observed by Calladine [43], can be illustrated with the aid of a simple paradigmatic example. For this instantiation, a rod with three- with aligned hinges shown in Figure 1a,b is utilized. When the lengths of the two members forming this system are smaller (larger) than the equilibrium lengths in the aligned configuration, the structure exhibits one (two) stable configuration(s). In more general systems, a stable equilibrium tensegrity configuration can also be converted into an unstable equilibrium by reversing the sign of the prestress; e.g., by switching cables with bars [43].

One of the main obstacles to the fabrication of tensegrity metamaterials is ensconced in the different nature of their constituent elements. In fact, while bars can be easily additively manufactured, it is still a technological challenge to effectively AM thin cables. Fabricating lattice structures with the same nodal positions and connectivity of a tensegrity structure, but equipped with only thick (or moderately thick) struts, surmounts this fabrication barrier. Nevertheless, it leads to the introduction of bending stresses in the system. Bending can be mitigated by tapering the struts at the ends, or by using flexible hinges [44,45]. However, this design strategy does not allow the fabrication of perfect hinges.

In light of the above considerations, it is evident that the fabrication of bistable lattices with tensegrity architecture becomes particularly convenient when the structure can be equipped with bars (struts) only. The present study utilizes this design strategy, and employs the AM technique of multiphoton lithography (MPL) [46,47] to reveal that the fabrication of bistable lattices with tensegrity architecture and nanoscale features can be successfully achieved. It should be noted that MPL is a convenient fabrication technique with which to manufacture the structures examined in the present study, with the desired resolution, since they involve cylindrical struts with minimum features (cross-section radii) of 250 nm. It must be noted that MPL, when used in conjunction with post-processing procedures, such as pyrolysis and etching, can achieve a spatial resolution smaller than 100 nm [48]. The bistable mechanical response of the structures designed and fabricated in this work is investigated through compression and indentation tests of different modular assemblies of tensegrity prisms composed only of bar elements. The force-displacement curves exhibited by the examined structures show a softening type response along the equilibrium branch initiating from the primary stable configuration. Such a branch terminates with a snapping event that drives the system into the secondary stable configuration. The examined experimental behaviors lead us to conclude that the fabrication of multistable metamaterials using MPL is highly feasible. They pave the way to the future studies on the application of the systems studied in this work as novel mechanical actuators and sensor, to be employed for the focusing of mechanical waves in narrow regions of space [15,16,17,18], and the development of a new paradigm for non-destructive evaluation and structural health monitoring of materials and structures [49].

## 2. Materials and Methods

### 2.1. Obtaining Bistable Frameworks from Monostable Tensegrity Structures

As it was previously elucidated, simple bistable frameworks based on monostable tensegrity structures can be obtained through recourse to the benchmark system shown in Figure 1a,b. This system is defined as a first-order infinitesimal mechanism or prestress-stable mechanism. It can possess a selfstress state, in which the two elements are in tension, imparting first-order stiffness to the internal mechanism [50]. If the two elements are linearly elastic, the load-displacement relationship for a load acting along the mechanism can be approximated by a cubic polynomial. The slope in the origin (*tan α* in Figure 1a) is directly proportional to the axial selfstressing forces in the elements. By reversing the sign of selfstress, leading to the compression of two elements in the aligned configuration, it becomes unstable. Moreover, moving along the mechanism, two more stable configurations can be found wherein the two elements are unstressed. The load displacement relationship in this case is the standard bistable snapping curve shown in Figure 1b.

A prestress-stable tensegrity structure with just one independent selfstress state and one independent infinitesimal mechanism embosoms the exact same behavior. Several examples in two dimensions have been presented (e.g., the one in Figure 1c). Typical three-dimensional examples include: the classical triangular tensegrity prism (Figure 1d) [51]; tensegrity prisms with rigid polygonal bases [52]; the expanded octahedron (aka tensegrity icosahedron) [53] (Figure 1e); the *x*-towers and the needle towers built by Kenneth Snelson (Hirshhorn Museum & Sculpture Garden, Washington D.C., United States) [52] (Figure 1f). As the (monostable) tensegrity structure is displaced along the mechanism away from the equilibrium configuration, its bars get compressed while its cables get tensioned. Furthermore, as the corresponding lattice structure is displaced along the mechanism from the unstable equilibrium configuration, the compression of the compressed elements is decreased, whereas elements in tension sustain less tension, until an unstressed stable equilibrium is reached.

The procedure for designing a bistable structure can be provided as follows: (i) find a prestress-stable tensegrity structure with just one independent selfstress state and one internal mechanism; (ii) consider a configuration slightly displaced along the mechanism and realize it as a conventional framed unstressed structure; (iii) adopt a reduced-order model or a finite element model to simulate its bistable response under static loads; (iv) adjust its geometric and material properties to adapt it to fabrication methods and experimental conditions; and (v) perform experiments on fabricated structures to confirm the designed behavior. It must be noted that for the step (i) it is necessary to consider only tensegrities where the number of elements *e* and the number of nodes *n* satisfy Maxwell’s relation for isostatic systems: *n* = *e* + 3 for two-dimensional systems, and *n* = *e* + 6 for three-dimensional ones.

### 2.2. Double Tensegrity Prism to Design a Bistable Unit and Corresponding Assemblies

**Figure 2:**
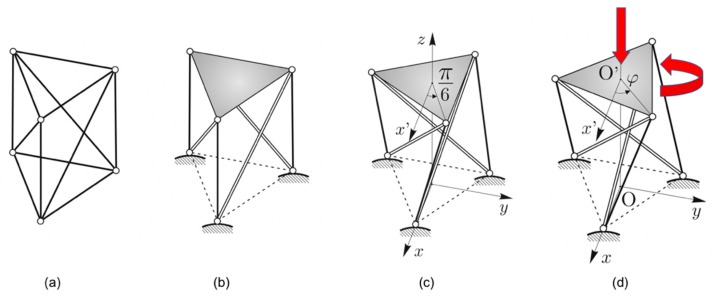
Design process of a tensegrity structure. (**a**) A bar framework in the shape of a regular right prism. (**b**) A corresponding right-handed tensegrity prism with cables (single line) and bars (double line). (**c**) Prestress-stable equilibrium configuration of the tensegrity prism. (**d**) When the internal mechanism is activated, the top triangle rotates about and translates along the vertical three-fold symmetry axis.

The standard triangular tensegrity prism shown in Figure 1b and Figure 2 is employed in the present work to design a bistable unit cell. This tensegrity structure can be obtained from a bar framework with the shape of a regular triangular prism and elements on the diagonals of the lateral faces of the prism (Figure 2a), such that the system possesses a three-fold symmetry axis passing through the centers of base triangles. The diagonal elements correspond to the bars, while all the others correspond to the cables of the tensegrity system, as shown in Figure 2b. The bottom nodes have been pinned to the ground and the bottom cables are removed, while the top triangle has been highlighted. Figure 2c shows the unique prestress-stable equilibrium configuration for a regular triangular prism, corresponding to a fixed value of its *twist angle φ*, which is the relative rotation between the two bases. For a tensegrity prism with triangular base, the twist angle is equal to *φ* = π/6. The internal mechanism of this system is a twisting motion, which is the combination of a vertical translation accompanied by a rotation of the top base with respect to the bottom base (Figure 2d). We define the *relative twist θ* = *φ* − π/6 to be the twist angle measured clockwise starting from the equilibrium configuration. For a tensegrity prism to be prestress-stable, it is only necessary to have *φ* = π/6, while the height of the prism and the sizes of each of the two equilateral polygonal bases have free rein. In addition, it can be observed that tensegrity prisms have a chiral geometry. The system shown in Figure 2 has a *right-handed* orientation, while its mirror image would have a *left-handed* orientation.

If a tensegrity prism is realized as a bar framework, and with a slightly smaller or larger twist angle, then it incurs a bistable behavior. Hence, it can snap from a primary stable configuration to a secondary stable configuration through a relative roto-translation between bases. Such motion is similar to the twisting mechanism of the parent tensegrity framework and will be referred to as a *bistable mechanism*. Each of the two stable configurations of such a bar framework is stress-free. During the activation of the bistable mechanisms, the diagonal elements are in tension, while the rest are in compression.

**Figure 3:**
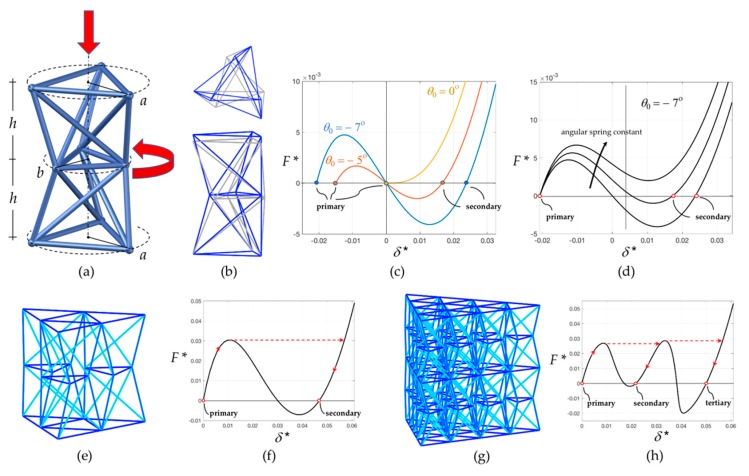
Mechanical modeling of the analyzed structures. (**a**) Geometry of the individual unit cell based on a double tensegrity prism. (**b**) Top view and side view of the primary (in blue) and secondary (in grey) stable configurations when the bistable mechanisms of the two prisms are activated simultaneously. (**c**) Static response of an individual unit cell with no angular springs under a vertical load for different values of the relative twist. (**d**) Static response of an individual unit cell with angular springs for different values of their spring constant. (**e**) Three-unit array. (**f**) Static response of a three-unit array. (**g**) Twenty-unit two-layer array. (**h**) Static response of a twenty-unit two-layer array.

The relative roto-translational motion between bases in a tensegrity prism is too perplexed to be used in practical bistable lattices. A simpler translational motion between bases can be obtained by superposing two tensegrity prisms with opposite orientations on top of each other, obtaining a *double tensegrity prism*. Figure 3a conveys the bar-framework corresponding to such a system, where each prism has height *h*; a small initial relative twist *θ*_0_; and different sizes *a* and *b* of base triangles. The doubled structure possesses two independent bistable mechanisms, which can be amalgamated together by composition to negate the relative rotation between end bases. When the top base is displaced vertically while keeping its rotation blocked, the bistable mechanisms of the two prisms are activated simultaneously, resulting in the rotation of the middle triangle only, as illustrated in Figure 3a. Figure 3b depicts the system in the corresponding stable equilibrium configurations before and after such process, which are addressed here as *primary* (in blue) and *secondary* (in grey). This system is the individual *bistable unit cell* which serves as a building block of the larger modular assemblies shown in Figure 3e,g. The size of the middle triangle in the bistable unit is selected such that there is enough clearance, circumventing collisions between adjacent units during activation of the bistable mechanism. While the single unit has two independent bistable mechanisms, assembling three unit cells side-by-side shown in Figure 3e leads to a single bistable mechanism. Correspondingly, a multi-layer assembly such as the one in Figure 3g has one bistable mechanism per layer.

A *Stick and Spring* reduced-order elastic model [54,55,56] is employed in this study to perform the numerical simulations of the structures. The nodal coordinates are selected as Lagrangian parameters. The bars are considered rigid with respect to bending and shearing deformations, and linearly elastic with respect to axial deformations. Linearly elastic angular springs are associated with variations in angle between the pairs of adjacent bars which are not part of a triangle of elements. These elements take into consideration the bending energy of an actual fabricated structure, assumed to be localized at the ends of the bars. Numerical calculations are performed in a regime of large displacements, taking into account geometric nonlinearities, following the approach provided in [54,55].

In a preliminary analysis of this unit cell, the circular cross section of bars has a radius of 375 nm, whereas the height of each prism is *h* = 7 μm, for a total height of the unit equal to 14 μm. The top and bottom base triangles are inscribed in a circle of radius *a* = 5 μm and the radius of the circle circumscribing the middle base triangle is *b* = 3.5 μm. The initial relative twist is *θ*_0_ = −7 degrees, corresponding to an initial twist angle *φ*_0_ = 23 degrees. The Young’s modulus is taken as equal to 1.2 Gpa (the methodology to obtain it is provided in the next subsection) and the stiffness constant of the angular springs has been assigned to be *k_s_* = 1.24 μN μm.

Each of the structures in Figure 3a,e,f have been subjected to a controlled-displacement uniaxial compression test, where the bottom nodes are kept fixed, while vertical downward displacements are imposed on the top nodes, without constraining horizontal displacements. Figure 3c displays the dimensionless force–displacement plot for the single unit (a) realized with no angular springs and different values of the initial relative twist angle *θ*_0_. The dimensionless force parameter *F* * is the resultant compressive force *F* divided by the axial spring constant of the shortest bar, *k_a_*, and by the bars’ diameter. The dimensionless displacement *δ* * is the actual vertical displacement divided by the unit’s height. The null value of *δ* * corresponds to the unstable equilibrium configuration.

When *θ*_0_ = 0, the response is qualitatively the same as the one of the parent tensegrity system. For negative values of *θ*_0_, the plot embosoms the form of typical bistable systems, with the snapping load increasing with the magnitude of the relative twist. Positive values of *θ*_0_ are impractical from an experimental viewpoint and are not considered in this work, as they correspond to an upward bistable mechanism which can be activated from the primary stable configuration by an upward displacement imposed on the top nodes. Figure 3d depicts the force-displacement plot for the single unit cell (**a**) realized with angular springs and for different values of the angular stiffness constant, keeping *θ*_0_ = −7 degrees. As the angular stiffness constant increases, the slope of the curve increases. In addition, if the force is never negative, the bistable behavior diminishes. Figure 3f elucidates the force-displacement relationship for a three-unit assembly (e): loading the primary configuration cause the structure to snap on the other equilibrium path; then the structure reaches the secondary equilibrium configuration upon unloading. Figure 3h presents the force-displacement relationship for a twenty-unit-two-layer assembly (g), where one of the two layers has slightly different spring constants. For this structure, two snapping events commence, one for each layer of the structure, and there can be three stable configurations.

### 2.3. Fabrication by Multiphoton Lithography and Mechanical Testing

**Figure 4:**
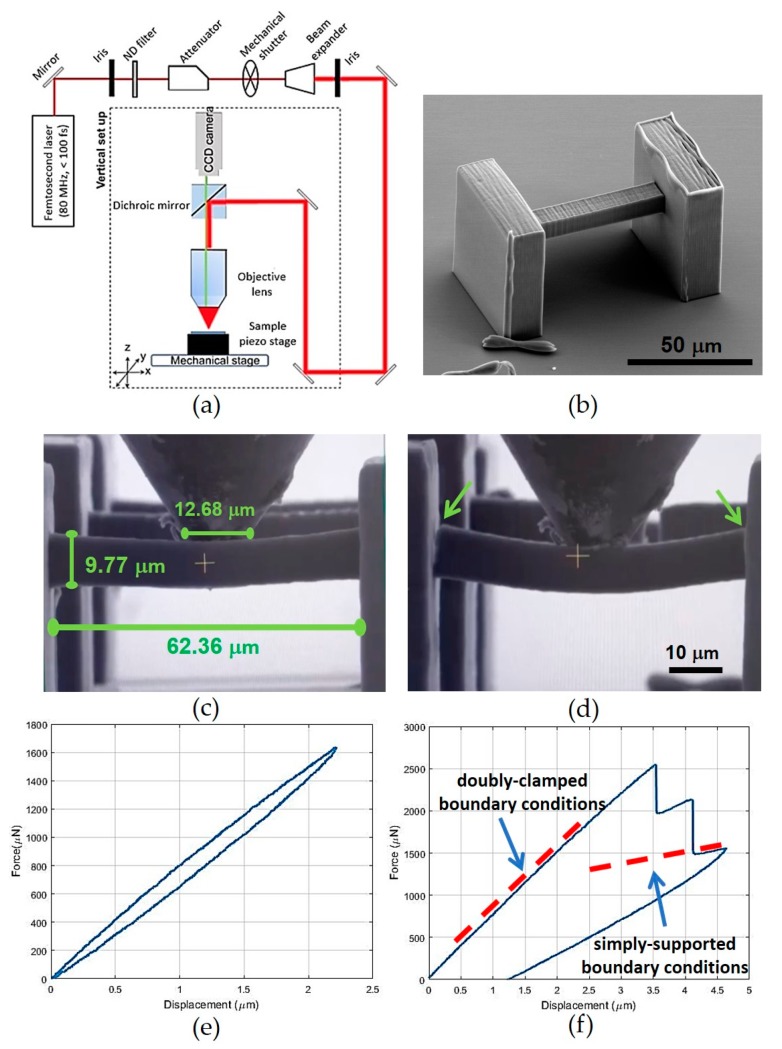
Experimental setup and testing to obtain the mechanical properties. (**a**) Schematic of the multiphoton lithography experimental setup [46]. (**b**) Beam structure employed for three-point bending measurements. (**c**) Beam structure at the beginning of the testing and dimensions. (**d**) Beam structure after cracking at the end sections. (**e**) A characteristic force-displacement curve obtained by three-point bending. (**f**) The force-displacement curve of a beam reaching the failure strength (cf. Video S1: Three-point bending).

All of the metamaterial structures analyzed in the present work were fabricated by multiphoton lithography [46] using the experimental setup shown in Figure 4a [57]. The structures were fabricated with a hybrid organic-inorganic material Zr-DMAEMA (FORTH, Heraklion, Greece) consisting of 70 wt% zirconium propoxide, 10 wt% (2-dimethylaminoethyl) methacrylate (DMAEMA) (Sigma-Aldrich, St. Louis, MO, United States) and 20 wt% ASTM type II deionized, distilled water. In total, 1.4 mL of 3-(trimethoxysilyl)propyl methacrylate (MAPTMS) (Sigma-Aldrich) was first mixed with 0.14 gr of hydrochloric acid in a vial. Afterwards, 137.7 μL of DMAEMA was mixed with 0.66 mL of zirconium (IV) propoxide solution containing 70 wt% 1-propanol in another vial. After adding the two mixtures together, they were diluted with 0.2 mL of distilled water and 0.016 gr of photoinitiator consisting of 4,4′-bis(diethylamino)benzophenone (Sigma-Aldrich). Before the fabrication, the experimental material was placed on glass substrates and remained for 24 h in vacuum. Further details regarding the material synthesis and experimental setup have been provided elsewhere [46]. It should be noted that multiphoton lithography is the only fabrication technique capable of constructing the minuscule structures examined in this study. All of the metamaterial structures were fabricated several times to ensure repeatability during both the fabrication and the mechanical testing.

In situ indentation tests were performed with a nanoindentation apparatus (PI 88 SEM PicoIndenter, Hysitron, Bruker, Billerica MA, United States) placed inside a scanning electron microscope ((Field Electron and Ion) FEI Quanta 3D (Field Emission Gun) FEG, FEI Company, Hillsboro OR, United States), thereby enabling high precision nanomechanical testing and real-time recording of the deformation. The molybdenum tip (model number 72SC-D3/035 (407A-M)) (Probing Solutions, Inc., Carson City NV, United States) was cut to a diameter of 70 μm by a femtosecond laser and then flattened using a focused ion beam. The maximum tip displacement was set at 10 μm. A triangular force function was used in all the tests. To ensure repeatability of the measurements, each experiment was repeated at least three times.

To obtain the mechanical properties of the photoresist employed for the fabrication of the structures, three-point bending was performed on single double-clamped beam members with square cross section. A characteristic structure is presented in Figure 4b, whereas the exact dimensions of the beam and the indenter are presented in Figure 4c. The side anchoring walls have dimensions 50 by 50 μm^2^ and thickness 20 μm.

For the uniaxial compression testing of the lattices, three different types of structures were fabricated, shown in Figure 1g–i. These are individual unit cells (Figure 1g), arrays of 3 unit cells in one layer (Figure 1h) and arrays of two layers with ten unit cells at each layer (Figure 1i).

**Figure 5:**
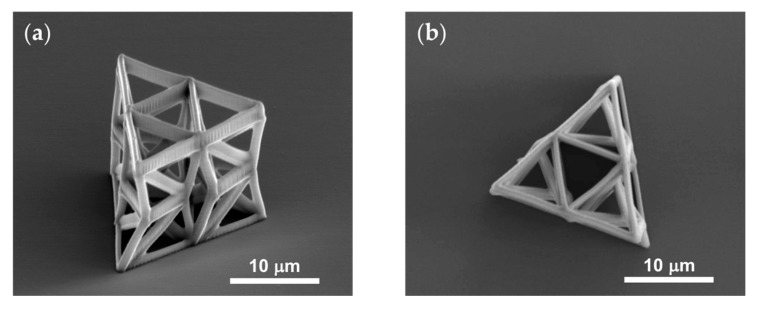
SEM images of a three-unit array. (**a**) axonometric and (**b**) top view.

Initially, individual unit cells were fabricated. The beams have an oval cross section with 250 nm and 500 nm minimum and maximum radii respectively. The dimensions of the individual unit cell are *h* = 7 μm, *a* = 5 μm and *b* = 3.5 μm, with an initial relative twist of *θ*_0_ = −7 degrees. After calibrating the fabricating conditions, the following geometric parameters were decided to be suitable for an efficient fabrications of the unit cell used in the arrays: *h* = 9 μm, *a* = 6 μm and *b* = 4.5 μm, and the initial relative twist *θ*_0_ = −9 degrees. All structures were fabricated with constant cross sections, since tapering of the cross section at the nodes resulted in inept photopolymerization of the structure. Figure 5 shows two SEM images of a fabricated three-unit array.

## 3. Results

### 3.1. Three-Point Bending of Double-Clamped Beams

Three-point bending tests were conducted as illustrated in Figure 4 on samples of the struts, by distributing the applied transverse load on the central region of the tested element (or beam). The force-displacement curve of a representative experiment is provided in Figure 4e,f. It is observed that the initial slope of the curve is 800 μN/μm, while the material failed at 2596 μN at deflection of the center of the beam at 3.545 μm. Given that the dimensions of the side anchoring walls are much larger than those of the beam cross section, such measurements are conducted under the assumption of a double-clamped beam. Consequently, the Young’s modulus was estimated using the Euler-Bernoulli theory equal to *E* = 1.281 ± 0.036 GPa and the breaking strength of the material equal to *σ*_Β_ = 131.99 ± 0.17 MPa. From the loading-unloading force-displacement plot in Figure 4e, a slight viscoelastic behavior of the material can be observed, characterized by hysteresis, energy dissipation and loading-rate dependency. In addition, from the plot in Figure 4f, two cracking events can be distinguished, highlighted by the two vertical drops in the plot. These are also confirmed by visual inspection of the images (Figure 4d) and the movie of the testing (see Video S1: Three-point bending). The cracking commences at the end sections of the beam, where the bending moment is maximum. This result is consistent with the assumption of clamped boundary conditions. After cracking, the beam can still sustain some loading, and the corresponding slope in the subsequent branch of the plot decreases to about one-fifth of the initial slope, a value which is consistent with simply supported boundary conditions.

### 3.2. Individual Unit Compression Testing

**Figure 6:**
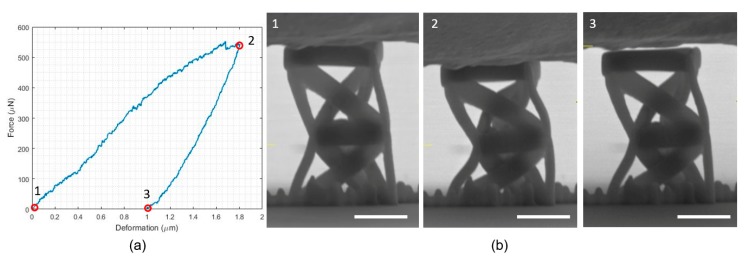
Mechanical testing on a single unit cell. (**a**) The force-displacement plot for an individual unit. (**b**) Snapshots of the structure at different times during testing (cf. Video S2: Individual unit). The white scale bar for each SEM figure is 5 μm.

Figure 6a shows the force-displacement plot obtained for the unit cell of the analyzed structures. The three snapshots of the unit cell in Figure 6b correspond to the three points highlighted in Figure 6a. Upon a visual inspection of the images and the movie of the testing (Video S2: Individual unit), it can be observed that the response of this structure is uneven, with the middle base of the unit moving out of the horizontal plane. This insinuates that the two prisms composing it may have different responses in the actual fabricated structure, and such a difference may be accentuated by the fact that an individual unit cell possesses two bistable mechanisms. However, despite this unsymmetrical behavior, twisting of the middle base during testing can still be observed. Furthermore, the loading branch of the force-displacement plot shows a softening tendency, while it is evident that there is a residual deformation after unloading. Both of these traits embrace the theoretical response of the elastic structural model. The force-displacement plot also reveals an initial slope during loading which is lower than the slope during unloading, consonant with the viscoelastic response and the polymeric nature of the bulk photoresist material.

### 3.3. Three-Unit Array Conpression Testing

**Figure 7:**
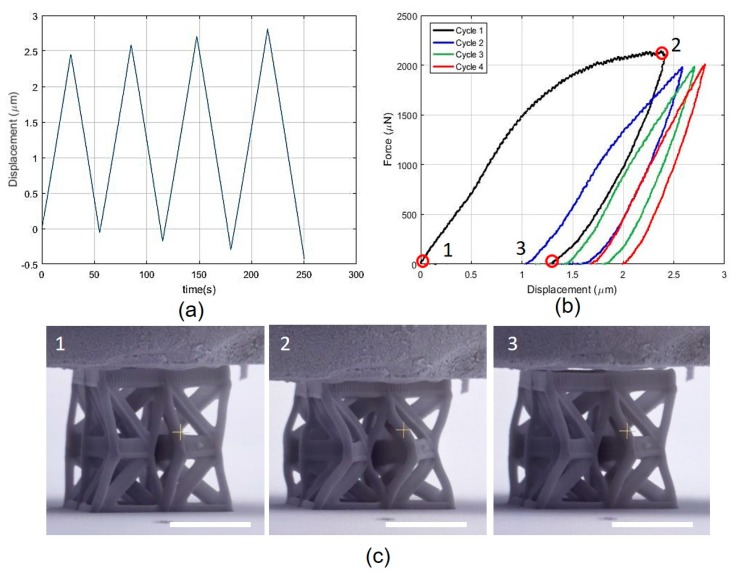
Mechanical testing on a three-unit array. (**a**) Imposed displacement vs. time and (**b**) force vs. displacement plots. (**c**) Snapshots of the sample during testing (cf. Video S3: Three-unit array). The white scale bar for each SEM figure is 8 μm.

Figure 7a,b shows the imposed displacement vs. time and the force vs. displacement plots for the array composed by three unit cells, while the snapshots shown in Figure 7c correspond to the points highlighted in Figure 7b. As a result of the adjustment and calibration of the fabrication process, and the utility of a different set of values for the geometric parameters of the unit cell, a smooth softening curve from point 1 to point 2 can be irrefutably observed. The curve starts with constant slope; then the slope increases slightly before decreasing smoothly until it becomes almost horizontal when it reaches point 2. At the first unloading, the slope of the curve is higher than the initial one, decreasing slightly until the force returns to a null level at point 3, when a new stable configuration is encountered. Inspection of the images and the movie of the testing (see Video S3: Three-unit array), reveals the discernible rotation of the middle triangles. In particular, the three middle triangles are free to rotate without interfering with each other and the twisting angle at the end of the first loading-unloading cycle differs from that of the initial configuration. Correspondingly, the height of the array at point 3 differs from the height at point 1 by approximately 1.2 μm. During the subsequent loading-unloading cycles, the response highlights a moderate preconditioning effect and a typical viscoelastic effect, while the twisting mechanism is preserved in the three units during each cycle.

### 3.4. Two-Layer Twenty-Unit Array Compression Testing

**Figure 8:**
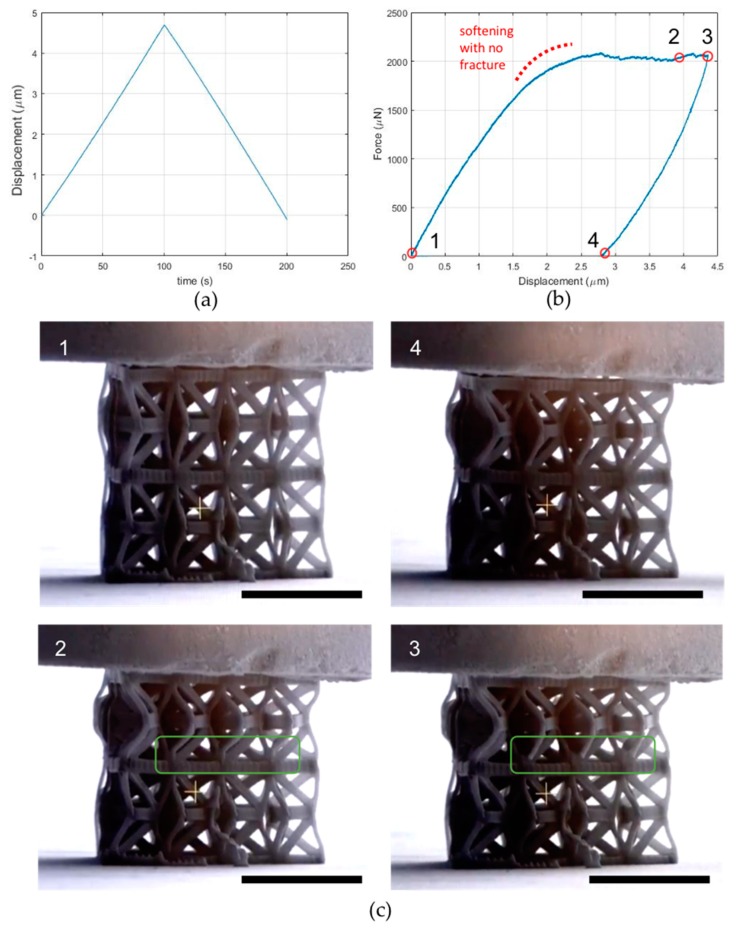
Mechanical testing on a twenty-unit two-layer array. (**a**) Imposed displacement–time and (**b**) force-displacement plots. (**c**) Snapshots of the sample during testing (cf. Video S4: Twenty-unit two-layer array). Some fracturing beams are enclosed in the green rectangle The black scale bar for each SEM figure is 10 μm.

Figure 8a,b shows the imposed displacement–time and the force-displacement plots for the array composed by two layers of ten units each, while the snapshots shown in Figure 7c correspond to the points highlighted in Figure 7b. The response of the structure is not as smooth as in the preceding case, with several irregularities in the plot which are likely to be related to microcracking events. Regardless, the softening response is still present, along with the residual deformation corresponding to the secondary stable configuration. Upon a visual inspection of the images and the movie of the testing (see Video S4: Twenty-unit two-layer array), the middle bases in the top layer are categorically rotating, while the rotation of those in the bottom layer is ensconced. This may be related to a higher stiffness of the bottom layer, which subsequently can be associated with the fact that the bottom layer is attached to the fabrication floor, and/or that there are undesired polymerized regions in the bottom layer.

### 3.5. Cracking and Fracture during the Testings

**Figure 9:**
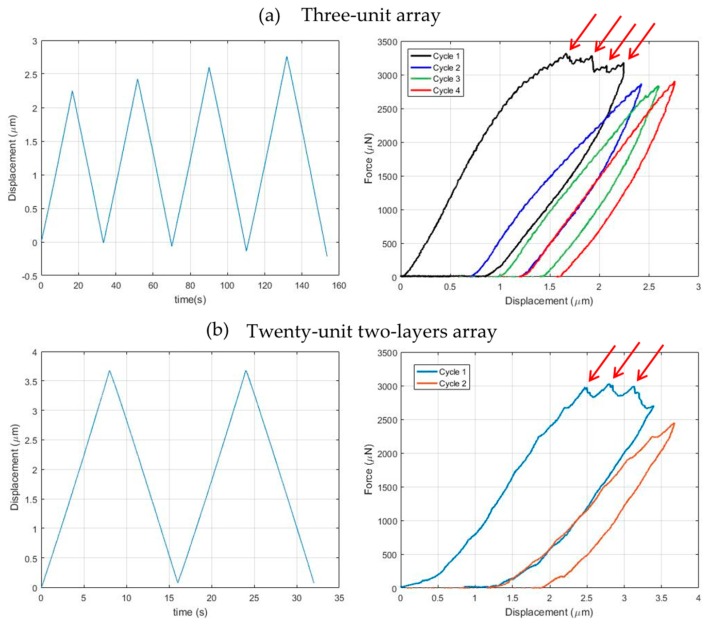
Mechanical responses revealing microcrack and fracture. (**a**) Imposed displacement-time and force-displacement plots for a three-unit array (cf. Video S5: Three-unit cracking). (**b**) Imposed displacement-time and force-displacement plots for a twenty-unit two-layer array (cf. Video S6: Twenty-unit two-layer cracking). Arrows indicate fracture events during the deformation.

**Figure 10:**
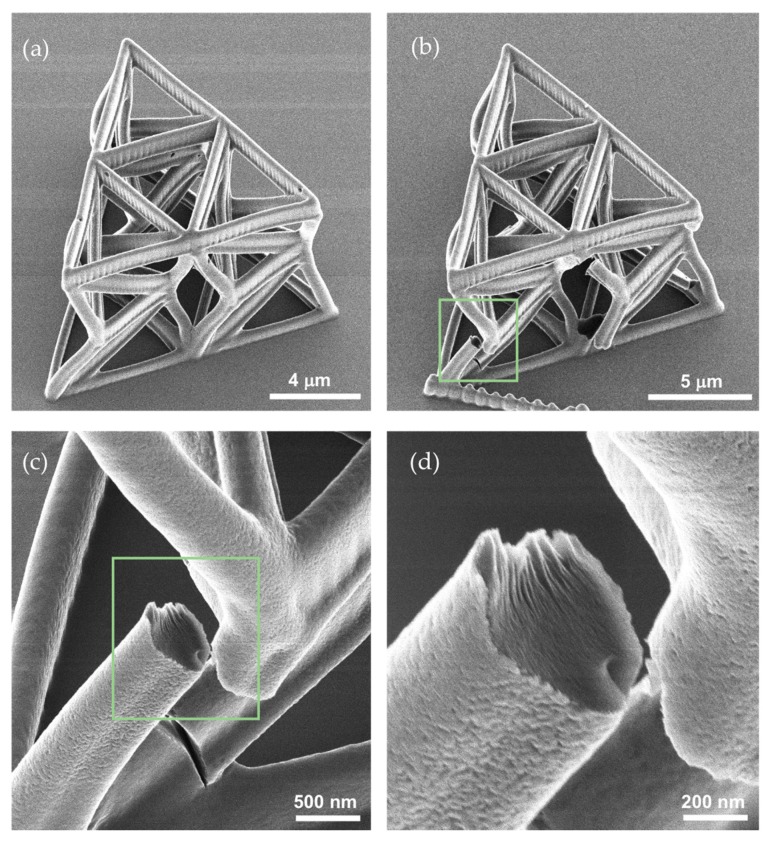
Helium ion microscopy (HIM) images of a three-unit array after testing. (**a**) HIM imaging of a structure that fracture did not commence. (**b**–**d**) HIM images of a fractured sample.

We observed fracture of the struts during several experiments. Figure 9 shows representative force-displacement plots and the corresponding images where fracture events can be observed (pointed at by the arrows in Figure 9). Upon a visual inspection, it is fairly clear that fracture occurred in beams because of excessive bending deformation. The breaking strength of the material is approximately 128 MPa. Considering for the sake of argument, a rectangular cross section with thickness 0.5 μm and width 1.0 μm, the radius of curvature of a beam at the onset of fracture, computed according to linear elasticity theory, is about 2.22 μm. This value seems to be consonant with the observed beam deformations. Figure 10 shows helium ion microscopy (HIM) images of the samples. HIM enables extremely high resolution (~20 nm features) avoiding sputtering of the samples, distorting their morphologies. Figure 10c,d shows the fractography. The dominant fracture mechanism is river markings, tacitly leading to brittle fracture.

## 4. Discussion

The main goal of this work was to address the question of whether lattice structures with nanoscale features and a bistable response can be efficiently fabricated through AM technologies. The experimental results given in Section 3 allow us to conclude that this objective can be satisfactorily accomplished utilizing the multiphoton lithography technique [46,47]. The design of functional models of 3D bistable tensegrities with nanoscale features required several iterative adjustments and modifications, as shown by the results presented in Section 2 and Section 3. A bistable-type response has been clearly observed in the two types of structures considered in this work, although in the two-layer system such a response was confined in space (cf. Section 3.4). Possible explanations of this behavior might be the following: (*i)* the bottom layer of such a system has a different stiffness with respect to the top layer; (*ii)* the bottom layer is contiguous with the fabrication substrate; (*iii)* there are undesired polymerized regions in the bottom layer.

Cracking and fracture of the tested samples often occurred during the execution of compression tests, when the imposed displacement caused excessive bending deformations in the beams/struts. The results presented in Section 3.5 highlight that such a drawback can be mitigated by taking the estimated value of the maximum curvature of the beams before failure as an input variable of the design procedure. The HIM images presented in that section show nanoscale river markings as fractography features, which indicate the occurrence of brittle fracture. These nanoscale features have never been reported before in the literature dealing with the fracture of nanolattice structures.

One important feature observed in the presented experiments was an appreciable viscoelastic behavior of the material composing the analyzed tensegrities, which was reflected in their experimental response under compression loading. Bistability is essentially a consequence of the geometrically nonlinear response of the examined tensegrity lattices. The force-displacement curves obtained under compression tests (Section 3.2, Section 3.3 and Section 3.4) highlight that bistable deformation mechanisms are combined with viscoelastic response in all the structures examined in the present work. Such an observation calls for the formulation of mechanical models accounting for viscoelastic response of tensegrity structures, which we address to future work.

Regarding the optimal design of novel bistable lattices with tensegrity architecture, it must be noted that the present work has established the theoretical basis of such a study. The required constraint of having just one independent state of selfstress and just one independent mechanism, (cf Section 2.1 and Section 2.2), can be easily implemented in available tensegrity form-finding procedures. This will accomplish bistable tensegrities with desired geometries [58,59,60]. While it is plausible to inquire for tensegrities with a target geometry, it must be observed that the problem of obtaining a desired snapping mechanism between two stable configurations is substantially challenging. Hence, it requires iterative design procedures that make use of accurate prediction models in the large displacement regime (cf. Section 2.2). A key feature revealed by the numerical results and the experimental tests presented in this work is that the analyzed structures exhibit large static indeterminacy. Consequently, despite the fact that each individual unit is actually an isostatic structure, the tested structures did not collapse even after the fracture of multiple struts (cf. Section 3.5).

**Figure 11:**
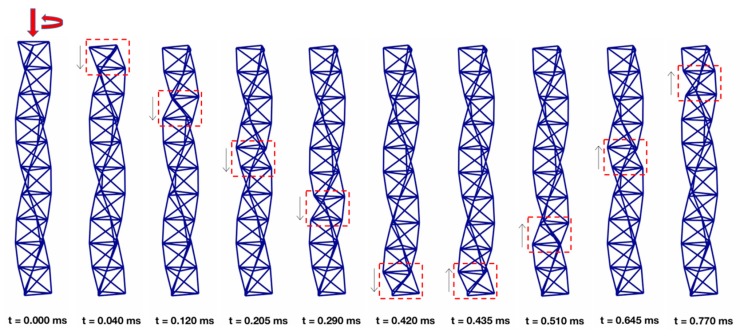
Snapshots extracted from the video of the motion of a column of ten bistable prisms impacted with initial vertical and angular speeds at the top base (see also Video S7 of Supplementary Materials).

Another peculiar property of a tensegrity system exhibiting a single soft mode is that the deformation process associated with the internal mechanism can be regarded as a “breathing,” or pumping motion, which can be efficiently employed to design novel types of bistable pumps at multiple scales [61]. Such a feature of bistable structures can also be exploited to design systems that support the transport of mechanical energy through compact solitary waves, which is a subject receiving growing interest in the area of nonlinear mechanical metamaterials [15,16,17,18,49]. Figure 11 conveys this characteristic quiddity of ten bistable prisms clamped at one end. Numerical results on the wave dynamics of such a system, which have been obtained through the procedures diffusely illustrated in [15,16,17,18,54,55], show that they support the formation of compression waves with compact support when subjected to an impulsive load at the free end.

Figure 11 shows some snapshots of the motion of the examined tensegrity column, which is impacted with initial vertical speed *v*_0_ and initial angular speed *ω*_0_ of the top base, so as to activate the bistable mechanism of the first unit. The simulations shown in Figure 11 correspond to assuming *a*/*h* = 0.5, *θ*_0_ = −3 deg, *v*_0_ (*E*/*ρ*)^−0.5^ = 0.3609, *v*_0_/*ω*_0_ = *a*^2^/*h, k_s_*/(*a*^2^ *k_a_)* = 0.0041, with *ρ* denoting the mass density of the material. One observes the propagation of a compression wave localized on a single prism (enclosed by the red dashed rectangle in Figure 11), with negligible motion of the rest of the column, under the examined loading condition. The reader is referred to the Video S7 of Supplementary Materials for an animation of the motion of the structure illustrated in Figure 11. The response of the benchmark bistable structure under examination highlights that the use of highly nonlinear tensegrity systems with nanoscale features may allow the creation of revolutionary types of acoustic lenses, to be used as a noninvasive scalpel to accurately target defects in engineering and biological materials. Micro- and nano-scale tensegrity lattices with bistable responses (acting as phononic crystals) can indeed be employed to generate compact-support waves within tensegrity acoustic lenses [18,49], which may travel and coalesce at a focal point in an adjacent medium (i.e., a material defect or a tumor mass in a host medium). A comprehensive study on this exciting, novel application of micro- and nano-scale tensegrites with a bistable response is addressed to future work. Furthermore, the structures analyzed in this paper utilize marked tunability (due to geometry and prestress) and scalability (size-independent properties) to go beyond conventional systems. The scalability property derives from the geometric nature of the bistable response, and the material nature of the viscous behaviors observed in the experiments. The mechanical modeling presented in this study can be applied down to the scale at which Van der Waals forces can be neglected, (several angstroms, see, e.g., [62], where tensegrity structures with strut length of 65 nm have been studied) A bistable viscous response can also observed in the macro-scale tensegrity structure shown in Video S8 of Supplementary Materials, which shows 20 cm timber struts connected with flexible polyvinyl chloride (PVC) tubes.

In addition, it must be noted that this work paves the way for the fabrication of tensegrity systems comprised of different polymeric materials. Even though this process requires specific photochemical properties to achieve such a high resolution [46], new materials must be employed that encompass hyperelastic behavior. Consequently, structures sustaining large deformations and repeatability of the loading under multiple cycles without failure could be realized.

## 5. Concluding Remarks

The bistable response of tensegrity structures made of three-dimensional assembles of tensegrity prisms was investigated in microscale structures equipped with nanoscale features. The modeling of the mechanical behavior of such structures provided guidelines for the fabrication of multi-cell systems featuring bistable responses under compression loading. MPL combined with diffusion-assisted high-resolution direct femtosecond laser writing enabled the efficient fabrication of unit cells and arrays comprised of struts with 250 nm radius. Microindentation experiments assisted by scanning electron microscopy imaging provided the in situ observation of nanoscale deformation phenomena and how they are reflected in the macroscopic force-displacement curves. Overall, the results presented in this work showed that the analyzed structures, which are comprised of all bar members, combine a bistable response; viscoelastic behavior; and softening and stiffening deformation mechanisms (cf. Section 3.1, Section 3.2, Section 3.3 and Section 3.4). In addition, helium ion microscopy elucidated the unblemished fractured morphology of the structures in nanoscale, insinuating brittle fracture as the primary fracture mechanism, regardless of the macroscopic ductile behavior (Section 3.5). These findings set the framework for the design and characterization of nanolattice structures governed by bistability for a variety of applications, with a particular focus on pioneering approaches to sound focusing and structural health monitoring through compact solitary waves [15,16,17,18,49].

## Supplementary Materials

The following are available online at https://www.mdpi.com/2079-4991/10/4/652/s1. Video S1: Three-point bending. Video S2: Individual unit. Video S3: Three-unit array. Video S4: Twenty-unit two-layer array. Video S5: Three-unit cracking. Video S6: Twenty-unit two-layer cracking. Video S7: Dynamic response of the column in Figure 11. Video S8: Bistable viscous response of a macroscopic tensegrity prism.

## References

[1] Liu Z., Zhang X., Mao Y., Zhu Y.Y., Yang Z., Chan C.T., Sheng P. (2000). Locally Resonant Sonic Materials. Science.

[2] Lu M.-H., Feng L., Chen Y.-F. (2009). Phononic crystals and acoustic metamaterials. Mater. Today.

[3] Maldovan M. (2013). Sound and heat revolutions in phononics. Nature.

[4] Brunet T., Leng J., Mondain-Monval O. (2013). Soft Acoustic Metamaterials. Science.

[5] Li H., Cheng G., Liu Y., Zhong D. (2020). Anomalous Thermal Response of Graphene Kirigami Induced by Tailored Shape to Uniaxial Tensile Strain: A Molecular Dynamics Study. Nanomaterials.

[6] Griffith A.S., Zhang T., Burkert S.C., Adiguzel Z., Acilan C., Star A., Saunders W.S. (2019). Characterizing the Cellular Response to Nitrogen-Doped Carbon Nanocups. Nanomaterials.

[7] Gan R., Fan H., Wei Z., Liu H., Lan S., Dai Q. (2019). Photothermal Response of Hollow Gold Nanorods under Femtosecond Laser Irradiation. Nanomaterials.

[8] Deng B., Mo C., Tournat V., Bertoldi K., Raney J.R. (2019). Focusing and Mode Separation of Elastic Vector Solitons in a 2D Soft Mechanical Metamaterial. Phys. Rev. Lett..

[9] Yildizdag M.E., Tran C.A., Barchiesi E., Spagnuolo M., Dell’Isola F., Hild F. (2019). A Multi-disciplinary Approach for Mechanical Metamaterial Synthesis: A Hierarchical Modular Multiscale Cellular Structure Paradigm. Green Nanomater..

[10] Meza L., Das S., Greer J.R. (2014). Strong, lightweight, and recoverable three-dimensional ceramic nanolattices. Science.

[11] Zheng X., Lee H., Weisgraber T.H., Shusteff M., DeOtte J., Duoss E.B., Kuntz J.D., Biener M.M., Ge Q., Jackson J.A. (2014). Ultralight, ultrastiff mechanical metamaterials. Science.

[12] Christensen J., Kadic M., Kraft O., Wegener M. (2015). Vibrant times for mechanical metamaterials. MRS Commun..

[13] Cummer S.A., Christensen J., Alù A. (2016). Controlling sound with acoustic metamaterials. Nat. Rev. Mater..

[14] Phani A.S., Hussein M.I. (2017). Dynamics of Lattice Materials.

[15] Fraternali F., Senatore L., Daraio C. (2012). Solitary waves on tensegrity lattices. J. Mech. Phys. Solids.

[16] Fraternali F., Carpentieri G., Amendola A., Skelton R.E., Nesterenko V.F. (2014). Multiscale tunability of solitary wave dynamics in tensegrity metamaterials. Appl. Phys. Lett..

[17] Davini C., Micheletti A., Podio-Guidugli P. (2016). On the impulsive dynamics of T3 tensegrity chains. Meccanica.

[18] Micheletti A., Ruscica G., Fraternali F. (2019). On the compact wave dynamics of tensegrity beams in multiple dimensions. Nonlinear Dyn..

[19] Shan S., Kang S.H., Raney J.R., Wang P., Fang L., Candido F., Lewis J.A., Bertoldi K. (2015). Multistable Architected Materials for Trapping Elastic Strain Energy. Adv. Mater..

[20] Raney J.R., Nadkarni N., Daraio C., Kochmann D.M., Lewis J.A., Bertoldi K. (2016). Stable propagation of mechanical signals in soft media using stored elastic energy. Proc. Natl. Acad. Sci. USA.

[21] Bilal O.R., Foehr A., Daraio C. (2017). Bistable metamaterial for switching and cascading elastic vibrations. Proc. Natl. Acad. Sci. USA.

[22] Chen T., Bilal O.R., Shea K., Daraio C. (2018). Harnessing bistability for directional propulsion of soft, untethered robots. Proc. Natl. Acad. Sci. USA.

[23] Deng B., Wang P., Tournat V., Bertoldi K. (2020). Nonlinear transition waves in free-standing bistable chains. J. Mech. Phys. Solids.

[24] Jeong H.Y., An S.-C., Seo I.C., Lee E., Ha S., Kim N., Jun Y.C. (2019). 3D printing of twisting and rotational bistable structures with tuning elements. Sci. Rep..

[25] Puglisi G., Truskinovsky L. (2000). Mechanics of a discrete chain with bi-stable elements. J. Mech. Phys. Solids.

[26] Guest S.D., Pellegrino S. (2006). Analytical models for bistable cylindrical shells. Proc. R. Soc. A: Math. Phys. Eng. Sci..

[27] Schioler T., Pellegrino S. (2007). Space Frames with Multiple Stable Configurations. AIAA J..

[28] Zirbel S.A., Tolman K.A., Trease B.P., Howell L.L. (2016). Bistable Mechanisms for Space Applications. PLoS ONE.

[29] Sajjad M., Makarov V., Mendoza F., Sultan M.S., Aldalbahi A., Feng P., Jadwisienczak W.M., Weiner B., Morell G. (2019). Synthesis, Characterization and Fabrication of Graphene/Boron Nitride Nanosheets Heterostructure Tunneling Devices. Nanomaterials.

[30] Pavlov D., Zhizhchenko A., Honda M., Yamanaka M., Vitrik O., Kulinich S.A., Juodkazis S., Kudryashov S.I., Kuchmizhak A.A. (2019). Multi-Purpose Nanovoid Array Plasmonic Sensor Produced by Direct Laser Patterning. Nanomaterials.

[31] Jipa F., Iosub S., Calin B., Axente E., Sima F., Sugioka K. (2018). High Repetition Rate UV versus VIS Picosecond Laser Fabrication of 3D Microfluidic Channels Embedded in Photosensitive Glass. Nanomaterials.

[32] Achour A., Islam M., Vizireanu S., Ahmad I., Akram M.A., Saeed K., Dinescu G., Pireaux J.-J. (2019). Orange/Red Photoluminescence Enhancement Upon SF6 Plasma Treatment of Vertically Aligned ZnO Nanorods. Nanomaterials.

[33] De Oliveira M., Wroldsen A.S. (2010). Dynamics of Tensegrity Systems.

[34] Oppenheim I.J., Williams W.O. (2000). Geometric Effects in an Elastic Tensegrity Structure. J. Elast..

[35] Oppenheim I.J., Williams W.O. (2001). Vibration of an elastic tensegrity structure. Eur. J. Mech.-A/Solids.

[36] Mascolo I., Amendola A., Zuccaro G., Feo L., Fraternali F. (2018). On the Geometrically Nonlinear Elastic Response of Class θ = 1 Tensegrity Prisms. Front. Mater..

[37] Pal R.K., Ruzzene M., Rimoli J. (2018). Tunable wave propagation by varying prestrain in tensegrity-based periodic media. Extreme Mech. Lett..

[38] Pajunen K., Johanns P., Pal R.K., Rimoli J., Daraio C. (2019). Design and impact response of 3D-printable tensegrity-inspired structures. Mater. Des..

[39] Micheletti A. (2013). Bistable regimes in an elastic tensegrity system. Proc. R. Soc. A: Math. Phys. Eng. Sci..

[40] Defossez M. (2003). Shape memory effect in tensegrity structures. Mech. Res. Commun..

[41] Xu X., Luo Y. (2010). Form-finding of nonregular tensegrities using a genetic algorithm. Mech. Res. Commun..

[42] Katz S., Givli S. (2018). Solitary waves in a bistable lattice. Extreme Mech. Lett..

[43] Calladine C. (1978). Buckminster Fuller’s “Tensegrity” structures and Clerk Maxwell’s rules for the construction of stiff frames. Int. J. Solids Struct..

[44] Lobontiu N., Paine J.S.N., Garcia E., Goldfarb M. (2000). Corner-Filleted Flexure Hinges. J. Mech. Des..

[45] Furqan M., Alam N. Finite element analysis of a Stewart platform using flexible joints. Proceedings of the 1st International and 16th National Conference on Machines and Mechanisms (iNaCoMM2013).

[46] Ovsianikov A., Viertl J., Chichkov B., Oubaha M., MacCraith B., Sakellari I., Giakoumaki A., Gray D., Vamvakaki M., Farsari M. (2008). Ultra-Low Shrinkage Hybrid Photosensitive Material for Two-Photon Polymerization Microfabrication. ACS Nano.

[47] Sakellari I., Kabouraki E., Gray D., Purlys V., Fotakis C., Pikulin A., Bityurin N., Vamvakaki M., Farsari M. (2012). Diffusion-Assisted High-Resolution Direct Femtosecond Laser Writing. ACS Nano.

[48] Seniutinas G., Weber A., Padeste C., Sakellari I., Farsari M., David C. (2018). Beyond 100 nm resolution in 3D laser lithography—Post processing solutions. Microelectron. Eng..

[49] Daraio C., Fraternali F. (2013). Method and Apparatus for Wave Generation and Detection Using Tensegrity Structures. U.S. Patent.

[50] Calladine C., Pellegrino S. (1991). First-order infinitesimal mechanisms. Int. J. Solids Struct..

[51] Micheletti A. (2004). Simple Analytical Models of Tensegrity Structures. Fracture Mechanics.

[52] Micheletti A. (2003). The Indeterminacy Condition for Tensegrity Towers: A Kinematic Approach. Revue Française de Génie Civil.

[53] Micheletti A. (2012). Modular Tensegrity Structures: The ”Tor Vergata” Footbridge. Fracture Mechanics.

[54] Favata A., Micheletti A., Podio-Guidugli P. (2014). A nonlinear theory of prestressed elastic stick-and-spring structures. Int. J. Eng. Sci..

[55] Amendola A., Favata A., Micheletti A. (2018). On the Mechanical Modeling of Tensegrity Columns Subject to Impact Loading. Front. Mater..

[56] Favata A., Micheletti A., Podio-Guidugli P., Pugno N.M. (2016). How graphene flexes and stretches under concomitant bending couples and tractions. Meccanica.

[57] Vangelatos Z., Komvopoulos K., Grigoropoulos C. (2018). Vacancies for controlling the behavior of microstructured three-dimensional mechanical metamaterials. Math. Mech. Solids.

[58] Micheletti A., Williams W. (2007). A marching procedure for form-finding for tensegrity structures. J. Mech. Mater. Struct..

[59] Kanno Y. (2013). Exploring new tensegrity structures via mixed integer programming. Struct. Multidiscip. Optim..

[60] Pietroni N., Tarini M., Vaxman A., Panozzo D., Cignoni P. (2017). Position-based tensegrity design. ACM Trans. Graph..

[61] Eberle P., Holler C., Müller P., Suomalainen M., Greber U.F., Eghlidi H., Poulikakos D. (2018). Single entity resolution valving of nanoscopic species in liquids. Nat. Nanotechnol..

[62] Liedl T., Högberg B., Tytell J., Ingber N.E., Shih W.M. (2010). Self-assembly of three-dimensional prestressed tensegrity structures from DNA. Nat. Nanotechnol..

